# Alcohol Advertising in Sport and Non-Sport TV in Australia, during Children’s Viewing Times

**DOI:** 10.1371/journal.pone.0134889

**Published:** 2015-08-11

**Authors:** Kerry S. O’Brien, Sherilene Carr, Jason Ferris, Robin Room, Peter Miller, Michael Livingston, Kypros Kypri, Dermot Lynott

**Affiliations:** 1 School of Social Sciences, Monash University, Melbourne, Australia; 2 Institute for Social Science Research, University of Queensland, Brisbane, Australia; 3 Melbourne School of Population and Global Health, University of Melbourne, Centre for Alcohol Policy Research, Turning Point, Fitzroy, Melbourne, Australia; 4 School of Psychology, Deakin University, Melbourne, Australia; 5 National Drug & Alcohol Research Centre, University of New South Wales, Sydney, Australia; 6 School of Medicine and Public Health, University of Newcastle, Newcastle, Australia; 7 Department of Psychology, Lancaster University, Lancaster, United Kingdom; University of Akron, UNITED STATES

## Abstract

Estimate the amount of alcohol advertising in sport vs. non-sport programming in Australian free-to-air TV and identify children’s viewing audience composition at different times of the day. Alcohol advertising and TV viewing audience data were purchased for free-to-air sport and non-sport TV in Australia for 2012. We counted alcohol advertisements in sport and non-sport TV in daytime (6am-8.29pm) and evening periods (8.30pm-11.59pm) and estimated viewing audiences for children and young adults (0–4 years, 5–13 years, 14–17 years, 18–29 years). During the daytime, most of the alcohol advertising (87%) was on sport TV. In the evening, most alcohol advertising (86%) was in non-sport TV. There was little difference in the mean number of children (0–17 years) viewing TV in the evening (N = 273,989), compared with the daytime (N = 235,233). In programs containing alcohol advertising, sport TV had a greater mean number of alcohol adverts per hour (mean 1.74, SD = 1.1) than non-sport TV (mean 1.35, SD = .94). Alcohol advertising during the daytime, when large numbers of children are watching TV, is predominantly in free-to-air sport TV. By permitting day-time advertising in sport programs and in any programs from 8.30pm when many children are still watching TV, current regulations are not protecting children from exposure to alcohol advertising.

## Introduction

Exposure to alcohol advertising is associated with initiation of drinking in children who have not previously been drinkers, and greater consumption in pre-existing drinkers [1–3]. In Australia, regulations stipulate that TV alcohol advertising should not occur at times when large numbers of children (0–17 years) are viewing, with 6am to 8.30pm typically considered peak viewing times for children [4–6]. However, clauses in the regulations allowing alcohol advertising in sport TV programming [6], undermine the protective intent of the regulations.

Greater restriction of alcohol advertising and sponsorship in TV programming has been recommended as a population-level strategy for delaying the initiation of drinking and reducing excessive alcohol consumption [7]. Bans or restrictions on alcohol advertising and sponsorship have been called for by peak medical and public health bodies in several countries (e.g., Australia, Ireland, UK, US, South Africa [8–12]). However, where governments have formally responded to such calls, it has often been to recommend that more evidence be gathered on the extent and nature of the problem before policy action can be considered [13].

A handful of studies have examined the extent of alcohol advertising on TV, but no research has established whether particular TV programs or genres (i.e., sport) have greater amounts of alcohol advertising, and at times when children may be watching. Similarly, few studies have conducted a nationwide analysis for a full year, establishing the number of alcohol advertisements at times when children may be watching [14,15]. Previous work in Australia has reported Target Audience Rating Points (TARPS), a marketing indicator of potential audience exposure to specific advertisements [16,17], finding high potential exposure of children to alcohol advertising. However, TARPS are not well understood by laypersons and policy makers, and do not provide absolute counts of alcohol adverts per se. Research reporting absolute alcohol advertising counts and audience numbers has been for brief periods of the year [18], potentially missing substantial seasonal variation, particularly in sport TV.

An analysis of alcohol advertising in sport is particularly important because the alcohol industry is thought to spend a considerable proportion of its advertising and sponsorship budget on sport TV programming [19–21]. Recent work examining references to alcohol in top class international and UK football found that there were 1.24 and 1.88 references to alcohol per minute, respectively [22,23]. Additionally, the alcohol industry and advertisers in some countries may exploit regulations whereby sport TV programming is allowed to contain alcohol advertising and sponsorship messages during children’s peak viewing times, but non-sport TV is not permitted to do so [5,6]. Such exemptions expose unknown, and possibly large, numbers of children to alcohol advertising while watching sport, nullifying any protective effect of restrictions [24].

The present study sought to establish the extent of alcohol advertising in free-to-air sport and non-sport TV programming in Australia in the daytime and night time for a calendar year. We also sought to estimate the proportion and number of young people in various age groups viewing TV at times when alcohol advertising is likely to be occurring.

## Methods

Information on all alcohol advertising in 2012 placed on the 11 commercial free-to-air TV channels in the five major metropolitan centres of Australia (Sydney, Melbourne, Brisbane, Adelaide, and Perth; 61% of the total Australian population) was purchased from Ebiquity an international media monitoring company (http://www.ebiquity.com). The data included the time/date, duration (seconds), program in which the advertisement was placed (e.g., sport vs. non-sport), and full content (i.e., video/sound/image). We did not examine subscription (pay-per-view) TV because it contains little commercial advertising and has low audience numbers relative to free-to-air TV [25].

We also purchased aggregated TV audience viewing data (i.e., time of day in ½ hour blocks) for all young people (age categories 0–4, 5–13, 14–17, 18–29 years) in the five major metropolitan centres from Australian Television Audience Measurement (OzTAM; http://www.oztam.com.au), the official source of television audience measurement in Australia (service provided by Nielsen Media Research). The OzTAM data allowed us to estimate viewing audiences in ½ hour slots by age-group to estimate potential alcohol advertising exposure between 6am and midnight for the free-to-air TV channels. While labour intensive, the analysis of advertising data from a complete 12 month period addresses limitations in previous work e.g., bias from monthly variation and across seasonal sport codes.

### Analysis

We report alcohol advertisement counts in sport and non-sport TV programming for three periods of the day: 6am to 8.29pm, 8.30pm to 11.59pm, and midnight to 5.59am (Table 1), and viewing audience numbers for each age category. Because the age categories vary in size (e.g., 5–13 = 9 years vs. 18–29 = 12 years), we also calculated adjusted proportions of viewers in each age category watching TV during the day (6am-8.29pm) and night (8.30pm-11.59pm). This involved a simple calculation where the number of viewers in each age category (see Table 2) is divided by number of years in each category (e.g., 5–13 years viewing at 6am-8.29pm = 117690/9 years = 13077). This adjusted viewing number was then divided by the sum of all age adjusted viewer numbers in respective age categories during the day 6am-8.29pm, yielding an adjusted proportion of viewers (13077/59262 = 22%). We also calculate the mean number of alcohol advertisements per hour in sport vs. non-sport TV (Table 3). ANOVA’s were used to examine whether there were significant differences in the mean number of alcohol advertisements per hour in sport vs. non sport TV for different periods of the day in the five metropolitan regions.

**Table 1:** Counts of alcohol advertisements in sport and non-sport TV programming by time in Australia’s five major metropolitan regions.

	6am-8.29pm		8.30pm-11.59pm		12am-5.59am		
City	Sport	Non-Sport	Sport	Non-Sport	Sport	Non-Sport	*X* ^*2*^ *P*-value*
Adelaide	653	98	529	2366	152	299	.0001
Brisbane	414	73	392	3115	114	389	.0001
Melbourne	664	84	582	2548	161	435	.0001
Perth	515	92	465	2892	192	335	.0001
Sydney	565	77	492	4348	159	736	.0001
TOTAL	2811	424	2460	15269	778	2194	.0001
%	86.9%	13.1%	13.9%	86.1%	26.2%	73.8%	

* *P*-values for Chi square analyses indicate significant differences in the counts of alcohol advertisements in sport vs. non-sport TV, for each of the times of day, for respective metropolitan regions.

**Table 2:** Average TV viewing audience per ½ hour time period by age and time.

Age in years	6am– 8:29pm	8:30pm– 11.59pm
0–4	117543	51048
5–13	117690	108417
14–17	38756	89548
18–29	155845	365679

Notes. The number of viewers is per ½ hour and may or may not be new/unique viewers. Audience viewing data from midnight to 5.59am was not available.

**Table 3:** Mean (*SD)* alcohol advertisements per hour of sport vs. non-sport TV programs that contained alcohol advertising for day vs. night-time for Australia’s five major metropolitan regions.

	6am-7.59pm		8pm-12am		
City	Sport	Non-sport	Sport	Non-sport	*P*-value*
Adelaide	1.82^a^	1.19^b^	1.72^a^	1.37^b^	.0001
	(1.11)	(0.64)	(1.10)	(0.73)	
Brisbane	1.64^a^	1.29^b^	1.63^a^	1.48^b^	.001
	(0.91)	(0.68)	(1.08)	(0.81)	
Melbourne	1.77^a^	1.22^b^	1.90^a^	1.42^b^	.0001
	(1.08)	(0.62)	(1.23)	(0.73)	
Perth	1.58^a^	1.32^a^	1.59^a^	1.51^a^	.07
	(0.82)	(0.70)	(0.94)	(0.83)	
Sydney	1.88^a^	1.15^b^	1.70^b^	1.49^b^	.0001
	(1.22)	(0.40)	(1.11)	(0.78)	
Overall	1.74^a^	1.23^c^	1.71^a^	1.46^b^	.0001
	(1.05)	(0.62)	(1.10)	(0.78)	

* Within rows, means with different superscript letters represent significant differences (at *P* < .01 level) in then mean number of alcohol advertisements in sport and non-sport programs in different day parts.

## Results

In 2012, there were 25,792 alcohol advertisements broadcast on all commercial free-to-air television channels across the five major Australian metropolitan areas. Program details were absent for 1856 advertisements (7%), which are not categorised within the sport vs. non-sport TV data reported here. The majority of the missing program details (62%) were for channel 7mate for Sydney.

For all categorised alcohol advertisements (23,936) for sport and non-sport TV for the five metropolitan regions, the majority (86%) were in non-sport TV between the hours of 8.30pm and 11.59pm (Table 1). From 6am till 8.29pm there was a reversal in the proportion of alcohol advertisements in non-sport (13%) vs. sport TV (87%). This pattern was similar across all metropolitan regions. Overall, Adelaide and Melbourne had the highest proportion of alcohol advertising in sport TV (32% and 31%, respectively), followed by Perth (26%), Brisbane (20%), and Sydney (19%). Differences in raw advertisement counts for sport vs. non-sport in different parts of the day for each metropolitan region were significant (see Table 1).

Table 2 displays the average number of viewers per ½ hour slot by time of day in each age category. The majority of the viewing audience between 6am and 8.29pm were children (N = 273,989), with the bulk (N = 235,233; 86%) below the age of 14 years. Between 8.30pm and 11.59pm, there were slightly fewer children viewing TV (average per ½ hour = 249,013) than between 6am and 8.29pm, and considerably more 18–29 year old viewers than in the earlier part of the day (365,679 versus 155,845).

Adelaide, Melbourne and Sydney had higher mean counts of alcohol advertisements per hour in sports TV than Brisbane and Perth (Table 3). Further, in Sydney there was a significantly higher mean number of alcohol advertisements per hour in sport TV earlier in the day than in any other genre at other times of day.

Collapsing across the metropolitan regions, the highest mean number of advertisements per hour was during sport TV between 6am and 7.59pm. Overall, there were significantly more alcohol advertisements per hour in a sport program during the daytime than in non-sport TV later in the day, which in turn was greater than non-sport TV earlier in the day.

### Alcohol advertising in televised sports and age of viewing audiences

**Fig 1:**
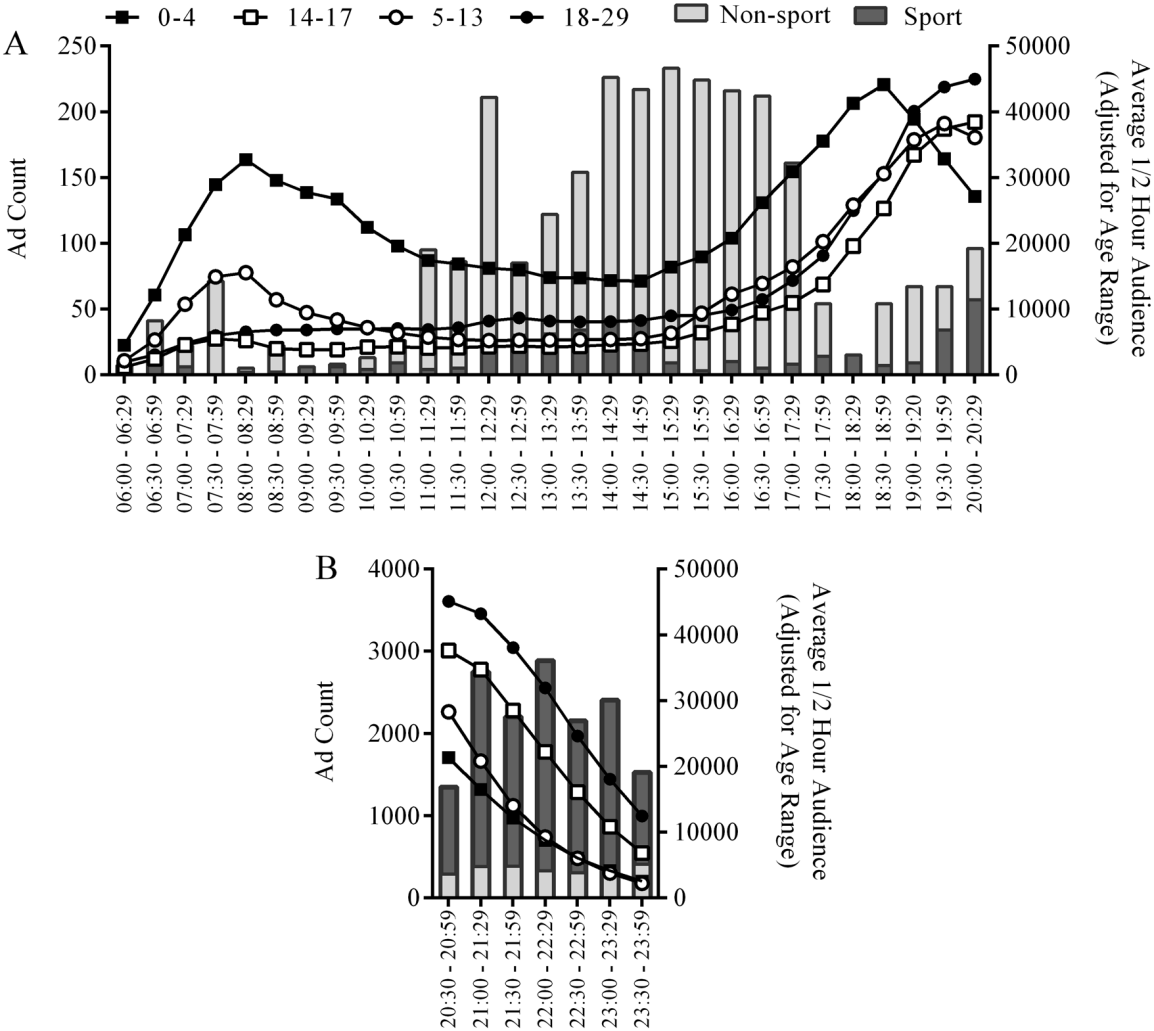
Alcohol advertising counts and children’s viewing audience per ½ hour. Bars represent total number of alcohol advertisements broadcast during sport and non-sport TV in each half hour time period for 2012 for the five metropolitan regions (left vertical axis) from (a) 6am to 8.29pm and (b) 8.30pm to 11.59pm, and lines represent the corresponding average ½ hour audience in each age category (adjusted for years in age range).

Overall, 25% of all alcohol advertising was in sport TV programming. Fig 1(a) shows the mean TV viewing audiences per ½ hour between 6am and 8.29pm by age category (adjusted for number of years in each), along with the number of alcohol advertisements in sport and non-sport TV during 2012. There was little difference in the adjusted proportion of viewers between the ages of 5 and 29 years (5–13 years: 22%, 14–17 years: 16%, 18–29 years: 22%). There were larger numbers of children aged 0–4 years (40%) watching TV at this earlier time. Between 8.30pm and 11.59pm (Fig 1(b)), there were more viewers aged 18 to 29 years than in other age categories. However, there were also large numbers of children viewing TV between 8.30pm and 11.59pm, with the slope of the reduction in viewing audiences similar across age categories. The adjusted proportions of young people viewing from 8.30pm till 11.59pm for each age category were 0–4, 14%; 5–13, 16%; 14–17, 30%; and 18–29, 41%.

Finally, Table 4 displays the number (and proportion) of advertisements for different alcohol products (e.g., beer, wine, spirits) within sport and non-sport programs. The majority of advertisements’ in both sport and non-sport TV were for beers, followed by spirits, ciders, wines, and premixed drinks.

**Table 4:** Number (proportion) of advertisements for different alcohol product categories within sport and non-sport TV programs.

Sport TV			Non-Sport TV	
	Count	(%)	Count	(%)
Beer	4120	68.11	10794	60.35
Spirit	813	13.44	2887	16.14
Cider	418	6.91	2151	12.03
Wine	342	5.65	1318	7.37
Pre-mix	356	5.89	737	4.12
Total	6049	100.00	17887	100.00

## Discussion

There were approximately 26,000 alcohol advertisements on Australian commercial free-to-air TV in 2012, an average of 71 per day. Between 6am and 8.29pm, a period when large numbers of children are watching TV, there were high counts of alcohol advertisements, with 87% placed in sport TV programming. The majority of alcohol advertisements were shown after 8.30pm, with 14% of alcohol advertisements in sport TV programming. The number of children watching TV during this later period was only slightly less than the number viewing in the earlier time period. Although this study cannot establish that children were paying attention to alcohol advertising at times they were judged to be watching TV, the results show that there is considerable potential for children to be exposed to alcohol advertising on free-to-air sport TV.

The results are consistent with the pattern of results from a previous analysis of TV alcohol advertising in the months of September and October 2010 which found 2810 alcohol advertisements in Australian free-to-air TV [18]. However, when extrapolated to a full year, the advertisement count reported in that study is lower than the estimate from the present study. This disparity may be due to October having a lower alcohol advertising count than other months, probably due to the lack of major sport competitions occurring in that month, with Australian rules football, rugby union, and rugby league having finished for the year and cricket yet to commence. Our findings are also consistent with the pattern reported by Fielder and colleagues [16], who examined potential exposure of children to alcohol advertising in Australia during 2005/2006. It is worth noting that there were a considerably greater proportion of alcohol advertisements in sport in Adelaide and Melbourne (31% and 32%, respectively) than in Sydney (19%). This difference appears to be due to a greater proportion of alcohol advertising being placed in Australian Football League (AFL) matches. AFL is the primary televised sport in Adelaide and Melbourne, but is less frequently played and televised in the Sydney metropolitan region. In Sydney, rugby league (NRL) is the dominant televised winter sports code, however, the NRL has less alcohol advertisements, instead relying more on alcohol branding on stadium signage, uniforms, and on pitch/group painted alcohol brands.

The extent of alcohol advertisements in sport TV is in line with US research showing that more than half of all beer advertisements were during sport programming [26]. In the present study we found that 68% of the advertisements in sport TV were for beers, though there were also a great proportion of beer advertisements (60%) in non-sport TV than other alcohol products. Madden and Grube [15] found that 77% of all beverage advertisements during prominent college and professional sport TV (i.e., football, basketball, baseball) were for beer. Furthermore, a recent detailed analysis of alcohol advertising in 20 sport and 20 non-sport TV programs in 25 US TV media markets found that 40% of youth exposure to alcohol advertising in these markets was via sport TV programming [13]. Advertising regulations in Australia permit alcohol advertising in non-sport TV between 12pm and 3pm on school days. The results show that between 12pm and 3pm, sport TV retained the majority share of alcohol advertisements.

Research suggests that alcohol advertising is more attractive to young people when presented in sport programming. For example, boys like beer ads more when placed within sport TV programming, and this greater liking is associated with stronger drinking intentions and higher alcohol use [27, 28]. Similarly, Grube and Wallack [29] found an association between viewing sport TV and awareness of alcohol advertising, which in turn was associated with more positive beliefs about alcohol.

Products presented within emotionally charged contexts are remembered, liked, and chosen more, even when the presentation of the products is not consciously processed by the viewer [30, 31]. Because sporting events are emotionally evocative for many viewers, they provide an ideal vehicle for advertisers to promote their products [32]. Sport TV programming is also known to attract the largest viewing audiences, both nationally and internationally. In Australia, 30 of the top 50 TV programs in 2012 were sport, with a cumulative viewing audience of 100 million people [33]. Further, the present research, along with other research on alcohol sponsorship of sport [34,35], suggests that sport is, to some extent, financially dependent on the alcohol industry. Such dependency is problematic when considering the wellbeing sport participants, fans, and the wider community. For example, Budweiser’s sponsorship of 2014 FIFA world cup resulted in Brazil having to change its laws to allow alcohol sales and consumption in sports stadiums [36]. The laws had previously been implemented to reduce high levels of alcohol-related violence and deaths. Accordingly, the interests of the alcohol industry may compromise the wellbeing of sport, fans, and the wider community.

There are some potential limitations of the present research. First, our analysis was restricted to the five major metropolitan regions of Australia. Although the majority of Australians live within these regions, the results may not generalise to smaller and rural regions. Second, individual sport and non-sport TV programs were each considered in aggregate, though there is probably considerable variation in the amount of alcohol advertising in different sports (e.g., football vs. swimming), and non-sport programs (e.g., situation comedies vs. documentaries). Third, because young people are increasingly using the internet to stream TV programming, which is not currently monitored in the same way as TV, the figures reported here probably underestimate audience numbers. Fourth, the advertising data reported here do not include advertising material placed on sport uniforms, stadia hoardings, or field signage, or in-game promotional comments regarding alcohol [23,24,37]. Finally, although there appear to be considerable numbers of children watching TV at times when alcohol advertisements are known to be aired, we cannot be sure that viewers were paying attention when the advertisement was played. Commercial data that could help more precisely estimate exposure to alcohol advertising (e.g., TARPS) for all of 2012 was prohibitively expensive, costing several hundred thousand dollars.

The results suggest it is likely that large numbers of children are frequently exposed to alcohol advertising on free-to air TV, and particularly through sport programming. This reflects limitations in advertising regulations that allow alcohol advertising (1) in sport TV during the day, and (2) at a time of night when large numbers of children are still watching TV. Accordingly, commercial advertising regulations do not appear to protect children from exposure to alcohol advertising.

Given that previous research showing that adolescent exposure to alcohol advertising is associated with earlier alcohol initiation and greater consumption [1–3], policy changes need to be made to reduce such exposure. The clause in advertising regulations whereby alcohol advertising is permitted in sport TV during children’s peak day-time viewing needs to be removed. There is no rationale for why sport programming during the day is allowed to contain alcohol advertising, when it is acknowledged that alcohol advertising is inappropriate in non-sport programming. Indeed, sport may enhance the influence of alcohol advertising on children and young adults [25, 26]. Because large numbers of children were found to be watching TV well after 8.30pm, policy makers need to revise current regulations in order to better protect children. The present data suggests that alcohol advertising should not be permitted in sport programming during the day time, and no alcohol advertising should appear on TV before 9.30pm. These simple measures would potentially halve children’s exposure to alcohol advertising.
