# Correction: Alcohol Advertising in Sport and Non-Sport TV in Australia, during Children’s Viewing Times

**DOI:** 10.1371/journal.pone.0139530

**Published:** 2015-10-02

**Authors:** Kerry S. O’Brien, Sherilene Carr, Jason Ferris, Robin Room, Peter Miller, Michael Livingston, Kypros Kypri, Dermot Lynott

There is an error in Fig 1, “Alcohol advertising counts and children’s viewing audience per ½ hour.” The color coding legend for the graphs is incorrect. Please see the corrected Fig 1 here.

**Fig 1:**
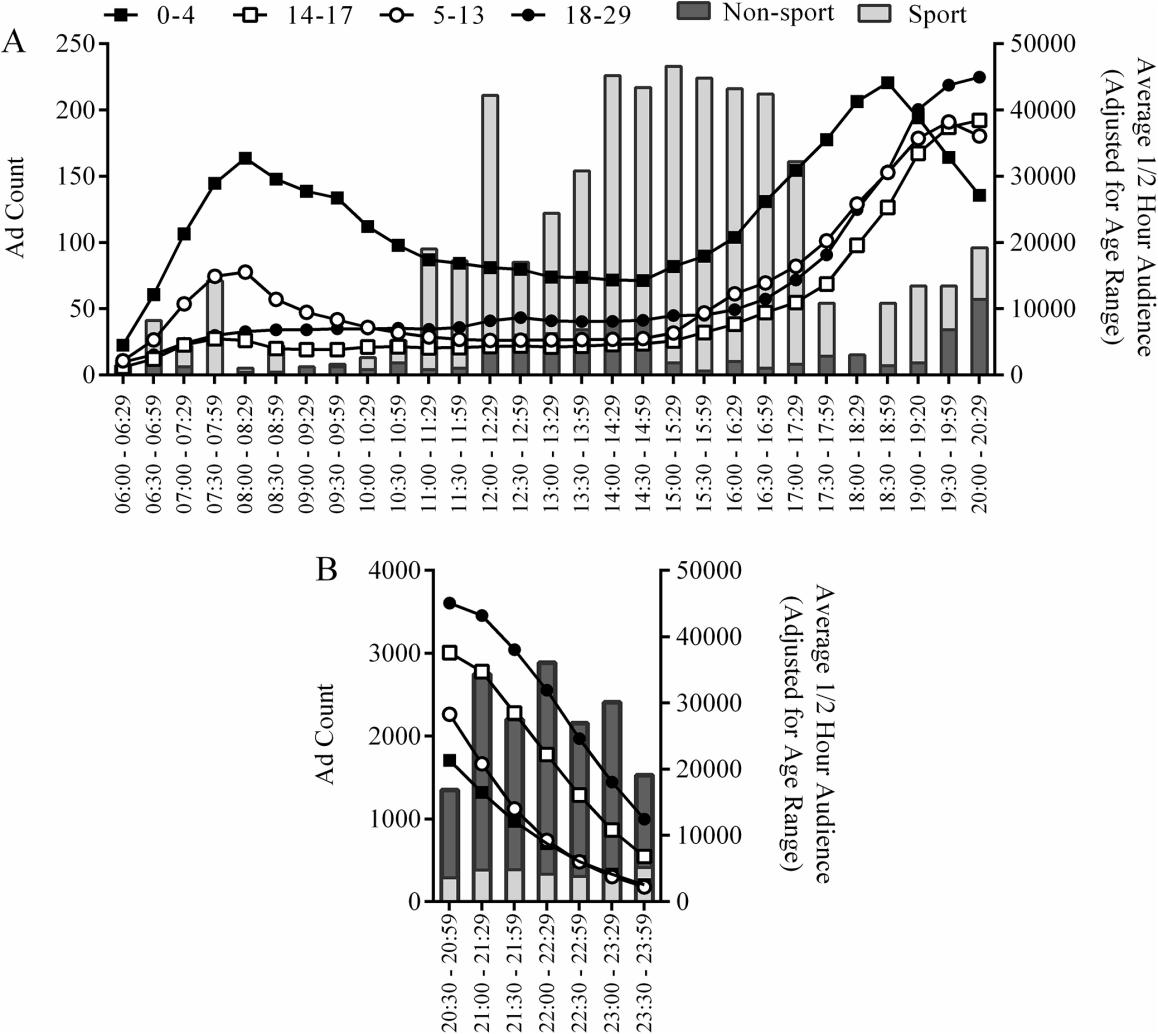
Alcohol advertising counts and children’s viewing audience per ½ hour. Bars represent total number of alcohol advertisements broadcast during sport and non-sport TV in each half hour time period for 2012 for the five metropolitan regions (left vertical axis) from (a) 6am to 8.29pm and (b) 8.30pm to 11.59pm, and lines represent the corresponding average ½ hour audience in each age category (adjusted for years in age range).
